# Evaluation of honey-baited FTA cards in combination with different mosquito traps in an area of low arbovirus prevalence

**DOI:** 10.1186/s13071-019-3798-8

**Published:** 2019-11-21

**Authors:** Nadja C. Wipf, Valeria Guidi, Mauro Tonolla, Michela Ruinelli, Pie Müller, Olivier Engler

**Affiliations:** 1 Department of Epidemiology and Public Health, Swiss Tropical and Public Health Institute, Socinstrasse 57, P.O. Box, 4002 Basel, Switzerland; 2 University of Basel, Petersplatz 1, P.O. Box, 4001 Basel, Switzerland; 3 Laboratory of Applied Microbiology, University of Applied, Sciences and Arts of Southern Switzerland, Via Mirasole 22a, 6501 Bellinzona, Switzerland; 4 Spiez Laboratory, Federal Office for Civil Protection, Austrasse, 3700 Spiez, Switzerland

**Keywords:** Arbovirus surveillance, *Culicidae*, Disease control, Mosquito-only flaviviruses, Nucleic acid preservation cards, Usutu virus

## Abstract

**Background:**

The threat of mosquito-borne diseases is increasing in continental Europe as demonstrated by several autochthonous chikungunya, dengue and West Nile virus outbreaks. In Switzerland, despite the presence of competent vectors, routine surveillance of arboviruses in mosquitoes is not being carried out, mainly due to the high costs associated with the need of a constant cold chain and laborious processing of thousands of mosquitoes. An alternative approach is using honey-baited nucleic acid preserving cards (FTA cards) to collect mosquito saliva that may be analysed for arboviruses. Here, we evaluate whether FTA cards could be used to detect potentially emerging viruses in an area of low virus prevalence in combination with an effective mosquito trap.

**Methods:**

In a field trial in southern Switzerland we measured side-by-side the efficacy of the BG-Sentinel 2, the BG-GAT and the Box gravid trap to catch *Aedes* and *Culex* mosquitoes in combination with honey-baited FTA cards during 80 trapping sessions of 48 hours. We then screened both the mosquitoes and the FTA cards for the presence of arboviruses using reverse-transcription PCR. The efficacy of the compared trap types was evaluated using generalized linear mixed models.

**Results:**

The Box gravid trap collected over 11 times more mosquitoes than the BG-GAT and BG-Sentinel 2 trap. On average 75.9% of the specimens fed on the honey-bait with no significant difference in feeding rates between the three trap types. From the total of 1401 collected mosquitoes, we screened 507 *Aedes* and 500 *Culex* females for the presence of arboviruses. A pool of six *Cx. pipiens/Cx. torrentium* mosquitoes and also the FTA card from the same Box gravid trap were positive for Usutu virus. Remarkably, only two of the six *Culex* mosquitoes fed on the honey-bait, emphasising the high sensitivity of the method. In addition, two *Ae. albopictus* collections but no FTA cards were positive for mosquito-only flaviviruses.

**Conclusions:**

Based on our results we conclude that honey-baited FTA cards, in combination with the Box gravid trap, are an effective method for arbovirus surveillance in areas of low prevalence, particularly where resources are limited for preservation and screening of individual mosquitoes.
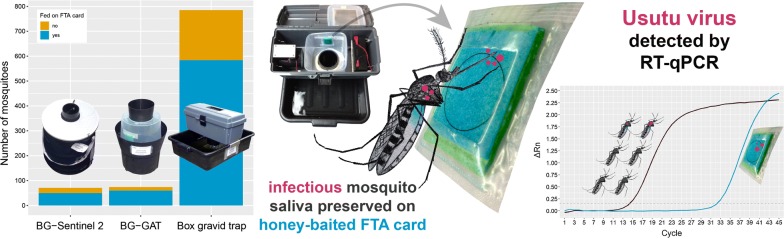

## Background

Arthropod-borne viruses (arboviruses) represent a serious public health problem as increasingly more viruses are (re-)emerging and spreading globally [1]. This trend is a consequence of growing global trade and travel activities, climate change and the high adaptability of viruses and their vectors [2, 3]. The devastating Zika virus (ZIKV) outbreak that struck the Americas in 2015 is the most recent example of how fast arboviruses can spread outside their endemic to other areas where mosquito vectors are already present and environmental conditions are suitable [4].

In Europe, the number of travellers returning with arboviral infections from endemic regions is increasing [5, 6]. At the same time the distribution of invasive mosquito species, competent to transmit these pathogens, is also expanding [7]. The threat of emerging arboviral infections in continental Europe is real, as demonstrated by several autochthonous chikungunya and dengue fever cases associated with the invasive Asian tiger mosquito, *Aedes albopictus* (Skuse). Of particular significance are two major chikungunya virus (CHIKV) outbreaks in Italy 2007 [8] and 2017 [9], each with more than 250 autochthonous human cases. In 2018 local transmissions of dengue virus (DENV) occurred in Spain with three confirmed cases and in southern France with six cases [10]. *Aedes albopictus* is the most probable mosquito vector responsible for all of these exotic virus transmissions on European mainland [7].

Despite extensive control efforts, the Asian tiger mosquito has also established stable populations in the south of Switzerland since its introduction in 2003 [11, 12]. Since then, the population density of *Ae. albopictus* in the Canton of Ticino (hereafter called Ticino) has presumably become sufficiently high to allow for local disease transmission [13, 14]. In addition, some indigenous mosquito species are also potential disease vectors. For example, *Culex pipiens* (*s.l.*) and *Cx. torrentium* are efficient vectors of West Nile virus (WNV) and are among the most abundant local mosquito taxa in Switzerland [15]. At the border to southern Switzerland in northern Italy, 173 autochthonous human neuroinvasive WNV cases were recorded between 2008 and 2015 [16].

Taken together, the co-occurrence of competent vector species and returning travellers infected with exotic viruses, as well as autochthonous transmissions to humans in neighbouring countries emphasises the necessity for a sensitive arbovirus surveillance method in Switzerland and the surrounding European countries. Typically, the circulation of arboviruses is noticed through reports of human cases, sentinel animals, entomological monitoring, or ideally through an integrated approach combining all three approaches [17].

Discovering an on-going arbovirus transmission through human or animal cases relies on the notification of these to the public health authorities. An important drawback of passive human case detection is that the majority of arboviral infections remain asymptomatic and thus unreported. Furthermore, the cases with disease manifestations might be underreported due to symptoms similar to other febrile illnesses or unavailability of unambiguous diagnostics.

Sentinel animals are immunologically naïve animals that are kept at strategic locations providing an early warning should they become infected with an arbovirus [18]. For this purpose, blood is drawn regularly from these sentinel animals and tested for the presence of virus-specific antibodies. The approach also comes with disadvantages, including lag time to answer, ethical concerns and unambiguous serological test results due to cross-reactivity with closely related viruses [19]. Additionally, currently most feared exotic viruses such as CHIKV, DENV and ZIKV exclusively infect humans and non-human primates and, therefore, may not be detected in chickens, pigs or horses that are the sentinel animals established in Europe.

As solely relying on autochthonous human or animal case detection means waiting until active transmission is already occurring, the preferable option is to perform surveillance in the virus-transmitting mosquitoes. Indeed, the detection of infected mosquitoes often precedes human or animal case detection, as has been shown for WNV in Italy [16]. Despite the urgent need for information on spatio-temporal occurrence of infectious mosquitoes to guide preventive actions, few European countries actually have a regular, integrated arbovirus surveillance system in place, partially because it is time- and cost-intensive. The commonly used mosquito-pool screening procedure involves laborious processing of thousands of mosquitoes requiring daily maintenance of collection traps and a constant cold chain to preserve viral RNA in freshly caught mosquitoes. Moreover, the proportion of infected mosquitoes in Europe is usually extremely low as shown in a review on WNV surveillance [14]. Therefore, particularly in areas of low transmission, a new entomological monitoring strategy that overcomes these limitations would be highly desirable.

An innovative technique, developed in Australia by Hall-Mendelin et al. [20] exploits the fact that infectious mosquitoes expectorate viruses in their saliva during sugar feeding. In their study mosquitoes were attracted to carbon dioxide (CO_2_) baited traps and were offered honey-soaked, nucleic acid preserving Flinders Technology Associates (FTA) filter paper cards within the trap chamber. They showed that viral RNA can be eluted and detected directly from the FTA cards by polymerase chain reaction (PCR), hence eliminating the time-consuming analysis of mosquitoes. The proprietary mix of chemicals on the FTA cards immediately inactivates viruses and other pathogens while preserving RNA and DNA for long-term storage at ambient temperature [21–23]. Australian researchers have been continuously using honey-baited FTA cards successfully for surveillance purposes [24, 25] and have even developed a sentinel mosquito arbovirus capture kit (SMACK) [26]. In contrast, a recent study that evaluated honey-baited filter papers to detect circulating WNV and equine encephalitis virus (EEV) in Florida, USA, found the approach to be the less sensitive method as compared to the already established sentinel chicken programme [27]. However, not FTA cards but different nucleic acid preserving substrates were used in this study.

The aim of our field study was to evaluate whether FTA cards are a sufficiently sensitive tool to detect potentially emerging mosquito-borne viruses in Switzerland, an area of low virus prevalence. Honey-baited FTA cards were used to collect potentially infectious saliva from mosquitoes in Ticino from July to October 2016. The first objective of the study was to evaluate the best trap type to be used in combination with the FTA cards by comparing three commercially available mosquito traps, including the BG-Sentinel 2, the BG-GAT and the Box gravid trap. The second objective was to evaluate the sensitivity of the new tool by testing both the collected mosquitoes and the corresponding FTA cards for the presence of mosquito-borne viruses.

## Methods

### Study area

We performed the field trial in urban and suburban areas of the 3 Ticino districts Locarnese, Luganese and Mendrisiotto from 13th July to 7th October 2016. In each district, we selected 4 municipalities: Gordola, Tenero, Minusio and Locarno in the Locarnese district; Pregassona, Lugano, Massagno and Paradiso in the Luganese district; Chiasso, Vacallo, Stabio and Mendrisio-Rancate in the Mendrisiotto district. In each of the 12 municipalities, we selected 3 trap positions, at least 100 m apart to avoid bias due to competition between traps. We arbitrarily selected the trap positions according to places where we would have expected an elevated risk for a potential transmission such as next to the hospital in Mendrisio, close to the asylum-seekers’ homes in Lugano, on camping grounds in Tenero and in public outdoor swimming pools in Locarno, Lugano and Vacallo. Additionally, in all 3 districts we placed traps in private gardens of residents who had reported an incidence of increased biting nuisance due to the Asian tiger mosquito.

The map of the study area was created using ArcGIS version 10.5 (ESRI Inc., Redlands, CA, USA) and with the Swiss base map from the Federal Office of Topography.

### Mosquito traps

**Fig. 1:**
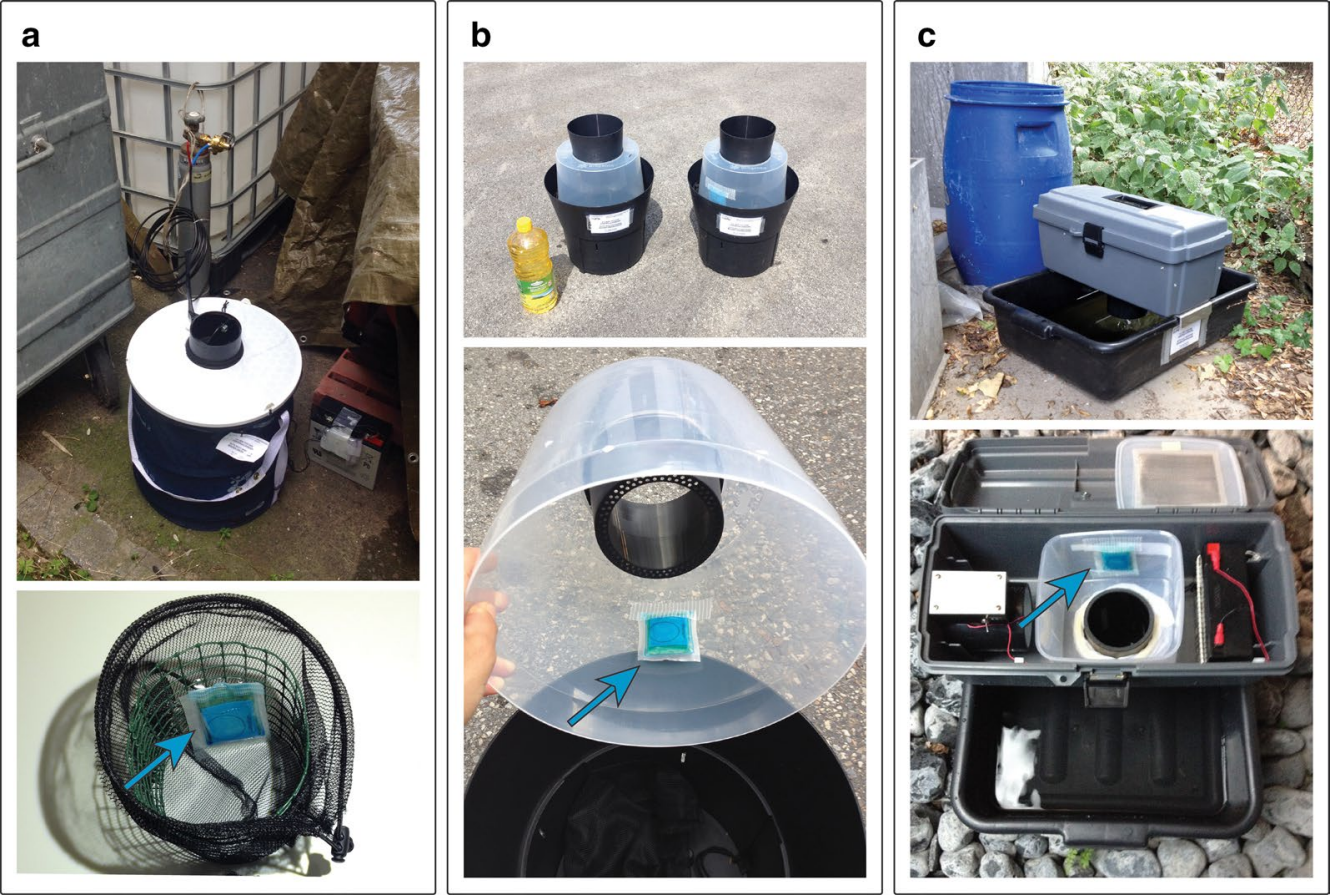
Mosquito traps used in combination with honey-baited FTA cards. The honey-baited FTA cards are indicated by blue arrows. **a** BG-Sentinel 2 trap baited with BG-Lure^®^ and CO_2_, image below shows the modified collection bag with the attached FTA card. **b** BG-GAT on the left wiped with canola oil without FTA card and BG-GAT on the right with an FTA card taped to the translucent chamber, shown below in the open state. **c** Box gravid trap, mosquitoes approaching the water surface are caught by the up-draft fan and sucked into the collection chamber where the FTA card was attached, shown below in open state

To assess the most suitable trap for collecting mosquitoes in combination with honey-baited FTA cards in Ticino, we measured the efficacy of three different mosquito traps side-by-side (Fig. 1). These traps included the BG-Sentinel 2 trap (Biogents, Regensburg, Germany), the BG-GAT (Biogents Gravid *Aedes* Trap, Biogents) and the Box gravid trap (BioQuip, Rancho Dominguez, CA, USA). We have chosen these traps because our aim was to catch mosquitoes that are competent for arboviruses, potentially circulating in Ticino. The target mosquito species were *Cx. pipiens*/*Cx. torrentium*, the main vectors for WNV and Usutu virus (USUV), and *Ae. albopictus*, an invasive species in Ticino where it is the only known potential vector for DENV, CHIKV and ZIKV, as well as an additional vector for WNV.

The BG-Sentinel 2 is designed to actively collect host-seeking female mosquitoes, primarily *Ae. albopictus* but also other taxa such as *Culex* species (Fig. 1a). The two attractants used for the BG-Sentinel 2 were BG-Lure^®^ (Biogents), imitating the odour of human skin and sweat, and CO_2_, mimicking the breath of humans or other vertebrates. The CO_2_ source was 3 kg of dry ice in a Styrofoam box for the first four weeks of the field trial and bottled CO_2_ with a flow rate of 70 ml/min for the remaining eight weeks. We modified the original collection bag by inserting a tube made of chicken wire with an attached FTA card (Fig. 1a). In a preliminary laboratory experiment we observed that the airflow generated by the BG-Sentinel 2 fan was too strong to allow trapped mosquitoes to feed on the FTA card. As the BG-Sentinel 2 comes with a shutter that automatically closes when the power is off we connected a timer module (Conrad Electronic SE, Hirschau, Germany) between the battery and the fan. We used lead-acid batteries (GS Yuasa, Kyoto, Japan) with 6 V and 10 Ah during the first four weeks of the field trial and with 12 V and 22 Ah for the remaining eight weeks. The timer was set so that the fan stopped periodically every other hour for one hour. This measure allowed the mosquitoes inside the bag to feed on the honey bait while the fan was stopped and the shutter closed.

The BG-GAT is designed to passively collect container-breeding mosquitoes without the requirement of a power source [28] (Fig. 1b). To increase its attractiveness, we baited the BG-GAT with 3 l of hay infusion [29, 30]. In the present study, we used two variants of the BG-GAT. In the first BG-GAT variant we taped a honey-baited FTA card to the untreated translucent chamber. In the second BG-GAT variant we wiped the translucent chamber with a film of canola oil but did not fit an FTA card. The manufacturer recommends applying oil to wet the wings of caught mosquitoes, preventing them from escaping. Unfortunately, it is impractical to tape an FTA card to an oil-treated surface and mosquitoes that are unable to fly could not approach the honey bait. While this was not an option for our purpose, we were still interested to know how the two variants of the BG-GAT compare with each other in terms of trapping efficiency. Therefore, we set the two trap variants in pairs while keeping a distance of at least 10 m between them.

The Box gravid trap is designed to attract gravid female mosquitoes searching for an oviposition site (Fig. 1c). Each Box gravid trap was baited with 4 l of hay infusion. Mosquitoes approaching the water surface were actively aspirated upwards into the collection chamber by a fan powered with a 6 V, 12 Ah, lead-acid battery (BioQuip).

With the exception of the BG-GATs with oil, each mosquito trap was equipped with a freshly prepared honey-baited Whatman^™^ non-indicating FTA Classic Card (GE Healthcare Life Sciences, Little Chalfont, UK) [20]. We cut the cards into quarters of 3.2 × 3.8 cm and left them overnight with the matrix area facing down on liquid honey (APIS Lebensmittel GmbH, Tenneck, Austria) that was coloured with blue-food dye (DEKOBACK GmbH, Helmstadt-Bargen, Germany) at a ratio of 100:1. The next morning, the honey-soaked FTA cards were placed individually into rectangular plastic sleeves that were welded together on three sides. The mosquitoes could feed on the matrix area of the FTA card through a rectangular opening. The opening was cut into the sleeve and was slightly smaller than the FTA card itself. To prevent the sugar solution from drying, a sponge moistened with blue-coloured honey-water mixture (1:10) was fitted behind the card.

### Sampling strategy

In total we had 36 trap positions, spread across 3 districts and 4 municipalities per district. Within a municipality we placed the traps at 3 positions. Each position had a different trap type, while the two BG-GAT variants were set in pairs. At each position, the traps remained for 48 hours, while we rotated the traps between trapping sessions so that at the end of the study each trap was at least twice at each position. After changing the battery type and CO_2_ source for the BG-Sentinel 2 trap, we repeated the complete first trapping round in the districts of Luganese and Mendrisiotto. The BG-Sentinel 2 that were set in the Locarnese district were equipped with dry ice and a 6 V, 10 Ah battery for the first full rotation, while they were equipped with bottled CO_2_ and a 12 V, 22 Ah battery for the second full rotation. In this field trial all four trap types completed 80 trappings of 48 hours each. Throughout the entire study we performed a total of 320 trappings, 240 of them with an FTA card. The detailed sampling schedule and the trap rotation scheme are provided in Additional file 1: Tables S1 and S2.

### Sample preparations

To avoid RNA degradation, and hence false negatives, we transported the mosquitoes in a cooler from the field to the laboratory in Bellinzona, where we killed them by freezing at −  20 °C. We then sorted the mosquitoes on a chilled metal plate according to their species using the morphological identification keys of Becker et al. [31] and Montarsi et al. [32]. We inspected each individual visually under a stereomicroscope (Leica EZ4 D, Leica Camera AG, Wetzlar, Germany) for signs of ingested blue-coloured honey. For each mosquito we recorded the collection date, trap position, trap type, species, sex and colour (blue or not) and then stored them at − 80 °C until RNA extraction. We removed the FTA cards from the plastic sleeves and stored them in individual ziplock bags at − 20 °C until molecular analysis. In the molecular analysis we tested both female mosquitoes and FTA cards for arboviruses in order to compare the two methods side-by-side.

### RNA extraction

We pooled the female mosquitoes from the same trap by species for *Aedes* and by genus for *Culex* specimens. Each pool was spiked with 10 μl of mengovirus culture of the vMC_0_ strain [33] to control for extraction efficiency and PCR inhibition. We homogenised each sample with a 5 mm stainless steel bead (Qiagen, Hilden, Germany) in 600 μl QIAzol lysis solution (Qiagen) using a TissueLyser II (Qiagen) at 30 Hz for 2 × 2 min. After adding an additional 300 μl QIAzol lysis solution, we extracted the RNA using the RNeasy Plus Universal kit (Qiagen) according to the manufacturer’s protocol. Final RNA elution of mosquito samples was done in 2 × 30 μl RNase-free water. We also spiked all FTA cards with 10 μl mengovirus culture as an external control and, following the procedures described by Ritchie et al. [24], we washed the FTA cards in 1 ml Whatman^™^ FTA Purification Reagent (GE Healthcare Life Sciences). Then, we purified the viral RNA from 560 μl of the eluate using the QIAamp Viral RNA Mini Kit (Qiagen) according to the manufacturer’s instructions. Finally, we eluted the RNA from the spin columns with 40 μl AVE Buffer. We stored all RNA extracts at − 80 °C until PCR analyses.

### PCR protocols

We analysed the extracted RNA templates from all mosquito pools and FTA cards with several PCR protocols targeting virus-specific sequences. To control for extraction efficiency and PCR inhibition we validated each sample with a mengovirus-specific reverse transcription real-time PCR (RT-qPCR) [34]. For the detection of alphaviruses and flaviviruses we ran endpoint reverse transcription PCRs (RT-PCRs), PanAlpha [35] and PanFlavi [36], with modified protocols (see below). We then sequenced the amplicons of samples that were positive in the RT-PCRs and confirmed them with a virus species-specific RT-qPCR if available. We included non-template and positive controls in every PCR run. All primers and probes are listed in Additional file 2: Table S3.

For the detection of mengovirus, the external control, we modified the RT-qPCR protocol of [34] as follows. Each 20 μl reaction contained 5 μl TaqMan^®^ Fast Virus 1-Step Mastermix (Thermo Fisher Scientific, Waltham, MA, USA), 0.4 μM of each the Mengo F2 forward and Mengo R2 reverse primer, 0.25 μM of Mengo P1 probe, and 4 μl of template RNA. We included extracted RNA from the same mengovirus strain that we used for spiking the samples as positive control on each RT-qPCR plate. We performed the RT-qPCR reactions in a 7500 Fast Real-Time PCR System (Applied Biosystems, Waltham, MA, USA), with the following thermal cycling conditions: 5 min at 50 °C, 20 s at 95 °C followed by 45 cycles of 3 s at 95 °C and 30 s at 60 °C.

For the nested endpoint RT-PCR targeting the non-structural protein 4 (nsP4) gene of alphaviruses [35] we used CHIKV RNA as the positive control. The PanAlpha RT-PCR consisted of a total reaction volume of 25 μl containing 5 μl 5× Qiagen OneStep RT-PCR Buffer, 1 μl Qiagen OneStep RT-PCR Enzyme Mix, 400 μM of the Qiagen dNTP Mix, 0.6 μM of each the Alpha 1+ forward and Alpha 1− reverse primer and 5 μl template RNA. Thermal cycling conditions were 30 min at 50 °C for reverse transcription, 15 min at 95 °C to inactivate the reverse transcriptase, for initial cDNA denaturation and activation of the DNA polymerase, followed by 45 cycles of 1 min denaturation at 94 °C, 1 min annealing at 50 °C and 1 min elongation at 72 °C, followed by a final extension step for 10 min at 72 °C. The subsequent nested PCR amplifications were carried out in a 20 μl reaction containing 10 μl 2× Qiagen Fast Cycling PCR Master Mix, 0.5 μM of each the Alpha 2+ forward and Alpha 2− reverse primer, and 1.5 μl of the PCR product from the previous reaction. The thermal cycling conditions were 5 min at 95 °C, followed by 45 cycles of 5 s at 96 °C, 5 s at 49 °C and 30 s at 68 °C, followed by a final extension step at 72 °C for 1 min.

For the semi-nested endpoint RT-PCRs targeting the non-structural protein 5 (NS5) gene of flaviviruses [36] we used ZIKV RNA as the positive control. The PanFlavi RT-PCR consisted of a total reaction volume of 25 μl containing 10 μl 2.5× Qiagen OneStep *Ahead* RT-PCR Master Mix, 1 μl 25× Qiagen OneStep *Ahead* RT Mix, 0.5 μM of each the MAMD forward and cFD2 reverse primer and 5 μl template RNA. Here, we used the following thermal cycling conditions: 10 min at 50 °C, 5 min at 95 °C followed by 40 cycles of 10 s at 95 °C, 10 s at 50 °C and 10 s at 72 °C with a final extension step for 2 min at 72 °C. The subsequent semi-nested 20 μl reaction contained 10 μl 2× Qiagen Fast Cycling PCR Master Mix, 0.5 μM of each the FS778 forward and cFD2 reverse primer and 1.5 μl of the 100-fold diluted PCR product from the previous reaction. The thermal cycling conditions were 5 min at 95 °C, followed by 45 cycles of 5 s at 96 °C, 5 s at 50 °C and 30 s at 68 °C, and a final extension step for 1 min at 72 °C.

We ran the PanAlpha and PanFlavi RT-PCRs in a Veriti AB Prism instrument (Applied Biosystems) and visualised the amplification products on 1.2% agarose gels stained with GelRed (Biotium, Fermont, CA, USA).

### Sequencing

We purified the endpoint RT-PCR products with Sephadex^®^ G-100 (Sigma-Aldrich, St. Louis, MO, USA) columns by centrifugation at 770×*g* for 3 min and then prepared the sequencing reactions with the BigDye^®^ Terminator v3.1 Cycle Sequencing Kit (Applied Biosystems). The sequencing reactions had a final volume of 10 μl containing 1 μl BigDye^®^ Terminator v3.1 Ready Reaction Mix, 1.5 μl 5× Sequencing Buffer, 0.2 μM of either the forward (FS778) or the reverse (cFD2) primer and 3 to 10 ng cDNA. The temperature profiles were as follows: 1 min at 96 °C, followed by 25 cycles of 10 s at 96 °C, 5 s at 50 °C and 4 min at 60 °C. We then purified the sequencing products with Sephadex^®^ G-50 (Sigma-Aldrich) columns by centrifugation at 770×*g* for 3 min and mixing with 5 μl of HiDi Formamide (Applied Biosystems) and sequenced the prepared cDNA in both directions on an Applied Biosystems 3500 Genetic Analyser (Applied Biosystems). We examined the sequences with the MEGA6 Software [37] and compared them against the BLAST Nucleotide database [38, 39].

### Data analysis

To evaluate the most efficacious trap type for mosquito collection in our study setting, we fitted the mosquito count data with a generalised linear mixed model (GLMM) with a negative binomial distribution and a log link function. Trap type was the fixed effect, while collection dates and trap position were included as random effects. To obtain a parsimonious model that fits the data well we aimed for (i) a low value for the Akaike’s information criterion; and (ii) a dispersion statistic close to one [40]. Additionally (iii) a plot of the Pearsonʼs residuals *versus* the fitted values without any obvious pattern was favoured. Similar to the mosquito counts, we analysed the feeding rates on the FTA cards by fitting a GLMM, while the negative binomial distribution was replaced by a binomial distribution and a logit link function. To test whether the trap type has a statistically significant effect on the feeding rate, we calculated *P*-values with the log-likelihood ratio tests between the model with and without trap type as a fixed effect term. The R code and GLMM outputs with explanations are provided in Additional file 3: Text S1. To test for differences in numbers of *Ae. albopictus* and *Cx. pipiens*/*Cx*. *torrentium* caught with each trap type we used Chi-square tests.

We performed the statistical data analysis with the freely available statistical software R, version 3.5.3 [41] in the integrated development environment RStudio [42]. For data cleaning and visualisation, we used packages of the “tidyverse” collection [43] and the GLMMs were fitted using the *lme4* package [44]. The level of significance was set at *α *= 0.05.

## Results

### Mosquito trap efficacy

**Fig. 2:**
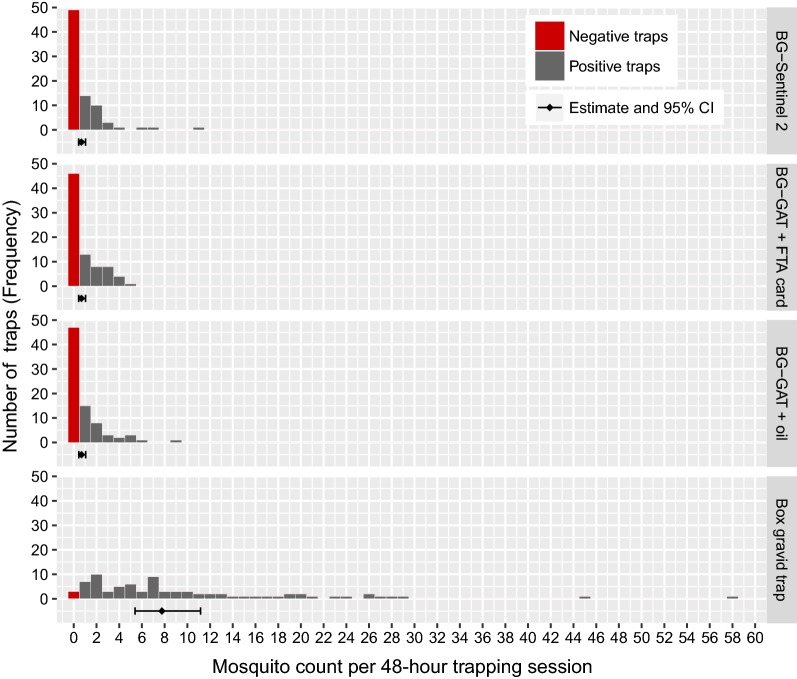
Comparison of mosquito trap efficacy. The histograms show the frequency of recorded mosquito count per 48-hour trapping session (*n *= 80 for each trap type) with red bars indicating negative traps. The diamonds and horizontal bars below represent the average mosquito count per 48-hour trapping session with 95% confidence intervals as estimated with the GLMM

For the purpose of arbovirus surveillance, we did only consider female mosquitoes of the genera *Aedes* and *Culex* being relevant in our study; and hence only these were included in the analyses that follow and are referred to as mosquitoes. The Box gravid trap was by far the most efficacious trap with 785 (77.9%) collected mosquitoes. The GLMM revealed that the Box gravid trap yielded significantly higher mosquito counts than the other traps (*P* < 0.001) (Fig. 2, Additional file 3: Text S1). The Box gravid trap caught on average 11.6 (95% confidence interval, CI: 8.0–16.8) times more mosquitoes than the BG-Sentinel 2. The difference in trapping success was neither statistically significant between the BG-Sentinel 2 and BG-GAT with an oil film nor between the BG-Sentinel 2 and the BG-GAT with an FTA card. More than half of the traps from these three trap types were negative, meaning they did not catch any of the mosquito taxa targeted for the arbovirus surveillance within a 48-hour period.

### Mosquito species composition

In total, the traps collected 1401 mosquitoes, 1015 (72.4%) females and 387 (27.6%) males from eight different taxa: *Ae. albopictus*, *Ae. geniculatus*, *Ae. japonicus*, *Ae. koreicus*, *Anopheles maculipennis* (*s.l*.), *An. plumbeus*, *Cx. hortensis* and *Cx. pipiens*/*Cx. torrentium*. *Culex pipiens* and *Cx. torrentium* cannot be distinguished morphologically; and hence are considered here as one taxon. As mentioned above, only female *Aedes* and *Culex* mosquitoes were included in the analyses; however, detailed information on all collected specimens, including males and *Anopheles* species, are given in Additional file 4: Table S4 and Figure S1 and Additional file 5: Table S5.

The Box gravid trap captured all of the identified species, while *Ae. albopictus* (*n *= 301; 38.3%) and *Cx. pipiens*/*Cx. torrentium* (*n *= 426; 54.3%) were the most prevalent taxa (Table 1). While the BG-Sentinel 2 caught almost equal numbers of the target species *Ae. albopictus* (*n *= 36) and *Cx. pipiens*/*Cx. torrentium* (*n *= 35) (*χ*^2^= 0.01, *df *= 1, *n *= 71, *P *= 0.91), the BG-GAT traps caught significantly more *Ae. albopictus* than *Cx. pipiens*/*Cx. torrentium* (BG-GAT with FTA card: *χ*^2^= 37.56, *df *= 1, *n *= 72, *P* < 0.01; BG-GAT with oil: *χ*^2^= 51.95, *df *= 1, *n *= 74, *P* < 0.01) and the Box gravid trap caught significantly more *Cx. pipiens*/*Cx. torrentium* than *Ae. albopicus* (*χ*^2^= 21.49, *df *= 1, *n *= 727, *P* < 0.01).

**Table 1:** Species composition of mosquitoes relevant for arbovirus surveillance in Ticino in 2016

Species	Total no. of females (%)	Total no. of females per mosquito trap (%)			
		BG-Sentinel 2	BG-GAT with FTA card	BG-GAT with canola oil	Box gravid trap
*Ae. albopictus*	467 (46.3)	36 (50.7)	62 (83.8)	68 (87.2)	301 (38.3)
*Ae. geniculatus*	1 (0.1)	0	0	0	1 (0.1)
*Ae. japonicus*	25 (2.5)	0	2 (2.7)	3 (3.8)	20 (2.6)
*Ae. koreicus*	14 (1.4)	0	0	0	14 (1.8)
*Cx. hortensis*	23 (2.3)	0	0	0	23 (2.9)
*Cx. pipiens*/*Cx*. *torrentium*	477 (47.3)	35 (49.3)	10 (13.5)	6 (7.7)	426 (54.3)
Unidentified	1 (0.1)	0	0	1 (1.3)	0
Total	1008	71	74	78	785

*Notes:* Absolute numbers (and percentages) of females captured in the entire study and with each trap type

### Sugar-feeding rates on honey-baited FTA cards

**Fig. 3:**
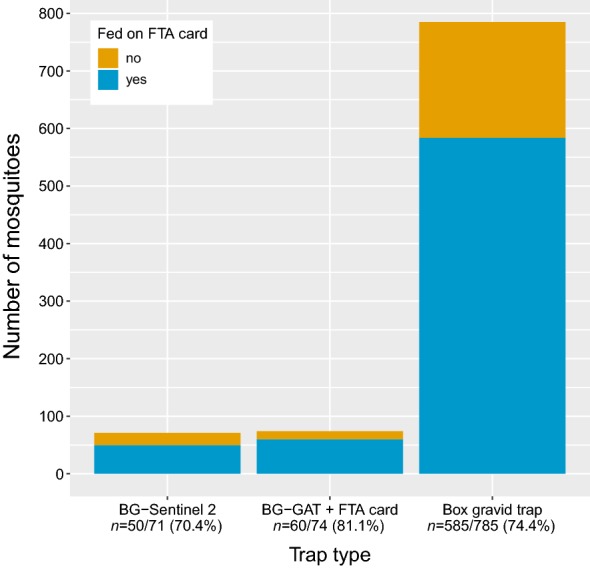
Comparison of sugar-feeding success between trap types. Blue bars represent the cumulative number of mosquitoes that fed on the honey-baited FTA cards in each trap type. On average 75.9% (95% CI: 70.8–80.4%) of the captured females fed on honey-baited FTA cards. There was no significant difference in sugar-feeding rates between the three trap types (*χ*^2^= 3.2, *df *= 2, *P *= 0.198)

As the honey on the FTA cards was coloured with blue food dye we could visually screen the specimens for individuals that had a honey meal. We observed no significant difference in sugar-feeding rates between the target species (*χ*^2^ = 2.38, *df* = 1, *n* = 870, *P* = 0.12). For each trap type the number of honey-fed mosquitoes outweighed the number of unfed mosquitoes with the Box gravid trap yielding the overall highest number of fed mosquitoes (Fig. 3). The GLMM for the feeding rate predicted that on average 75.9% (95% CI: 70.8%–80.4%) of the mosquitoes feed on honey-baited FTA cards, while there was no significant difference in feeding rate between the three trap types (*χ*^2^= 3.2, *df *= 2, *P *= 0.198). In the Box gravid trap an average of 6.7 (95% CI: 5.2–8.7) mosquitoes fed on an FTA card, while much fewer honey-fed individuals could be retrieved from the other trap types. In the BG-Sentinel 2 on average 1.4 (95% CI: 0.9–2.2) mosquitoes and in the BG-GAT 1.6 (95% CI: 1.0–2.3) were honey-fed after the 48-hour trapping period. See Additional file 3: Text S1 for the GLMM and Additional file 6: Figure S2 for the histogram and average number of mosquitoes that fed on FTA cards. The sugar-feeding success of all female and male mosquitoes caught in our study is summarised in Additional file 6: Table S6.

### Virus detection

We analysed a total of 265 mosquito pools, comprising 1008 females (507 *Aedes*, 500 *Culex* and one individual with unidentified species), and 240 FTA cards for the presence of viruses. We spiked all mosquito pools and FTA cards with mengovirus culture as an external control. Reverse transcription qPCRs targeting mengovirus RNA were positive for all samples, indicating successful RNA extractions and absence of PCR inhibitors. We screened the mosquitoes and FTA cards for both alphaviruses and flaviviruses by endpoint RT-PCR and then sequenced any positive sample. All samples were negative for alphaviruses. However, for three mosquito pools and one FTA card we detected a flavivirus-specific band with the PanFlavi RT-PCR.

**Fig. 4:**
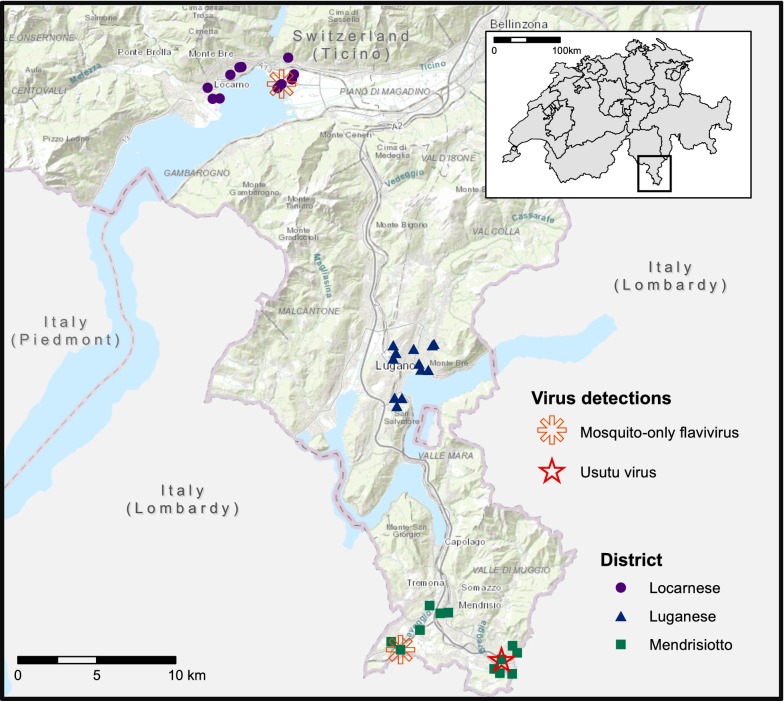
Map of sampling area in the Canton of Ticino, southern Switzerland. Each filled symbol represents one of the 36 trap positions. Unfilled symbols indicate virus detections at the underlying trap position. Source of base map: Swiss Federal Office of Topography, swisstopo

From one of these three flavivirus-positive mosquito pools we were able to sequence USUV. It is noteworthy that this USUV-positive pool consisted of six *Cx. pipiens*/*Cx*. *torrentium* and in two of these specimens we detected blue honey by visual inspection. Intriguingly, the FTA card from the same trap also tested positive for USUV. Pairwise alignment of the obtained sequences revealed that the USUV isolated from the *Culex* pool (GeneBank accession number: MN566102) was identical to the USUV isolated from the FTA card (GeneBank accession number: MN566103). According to the BLAST result the USUV found in our study shares the highest sequence similarity with USUV isolated from *Cx. pipiens* in northern Italy in 2010 [45]. For both the mosquito pool (Cq = 13.7) and the FTA card (Cq= 31.5), we could confirm the presence of USUV by means of the virus species-specific RT-qPCR. The amplification plot is shown in Additional file 7: Figure S3. The USUV-positive mosquitoes and FTA card were sampled with a Box gravid trap in the public swimming pool of Vacallo between 27th and 29th September 2016 (Fig. 4).

In addition to the USUV, two *Ae. albopictus* collections were positive for mosquito-only flaviviruses (MOFs) (Fig. 4). The BLAST results showed that both of our sequenced samples have the highest sequence similarity to mosquito flaviviruses likewise isolated from *Ae. albopictus*. We recovered the first MOF-positive sample from a pool of three *Ae*. *albopictus* from a BG-GAT trap with oil (i.e. without an FTA card) that was located in Tenero and placed between 7th and 9th September 2016 (GeneBank accession number: MN566100). The second MOF-positive sample was a single *Ae. albopictus* recovered from a Box gravid trap that was located in Stabio and sampled between 19th and 21st September 2016 (GeneBank accession number: MN566101). None of the FTA cards were positive for MOFs. The summary information of the four virus positive samples and the complete sequences are listed in Additional file 7: Table S7 and Text S2.

## Discussion

While the sugar-based FTA card surveillance approach has been proven useful in detecting circulating viruses in high transmission areas [20, 24–27, 46], the aim of this study was to test its suitability in combination with three different mosquito traps, the BG-Sentinel 2, the BG-GAT and the Box gravid trap, as a monitoring strategy to detect potentially emerging mosquito-borne viruses in Switzerland. We found that (i) we can confirm the presence of USUV in mosquitoes from FTA cards even in an area of low prevalence among the tested traps, and that (ii) the Box gravid trap was by far the most efficacious trap for sampling *Ae. albopictus* and *Culex* mosquitoes.

One mosquito pool and the FTA card from the same Box gravid trap were positive for USUV, an emerging virus in Europe that is closely related to other mosquito-borne flaviviruses such as the Japanese encephalitis virus and WNV. The virus is spread with infected birds as amplifying hosts and mosquitoes as vectors [47]. In Switzerland, USUV killed a considerable number of captive and wild birds in and around Zurich Zoo in 2006 [48]. In a study carried out in 2011 and 2012 USUV was found in mosquitoes from Ticino [14]. Although mainly pathogenic to birds, USUV may cause neuroinvasive infections in immunocompromised patients [49]. In 2009 two cases of USUV causing neurological disorders were recorded from neighbouring Italy [50, 51], highlighting the importance of detecting USUV circulation not only for veterinary but also for human health.

In addition to USUV, we also found an *Ae. albopictus* specimen being positive for MOF in a Box gravid trap equipped with a honey-baited FTA card. Mosquito-only flaviviruses are supposedly non-pathogenic to humans and animals [52, 53]. Even though the mosquito fed on the sugar-bait, MOF was detected neither from the FTA card in the same trap nor from any other FTA card. It is assumed that MOFs persist primarily through vertical transmission [54–56] and may, therefore, not be expectorated with saliva, which might explain why we could not detect the virus on any of the FTA cards. Likewise, in a Catalonian study an *Ae. albopictus* pool was found positive for MOFs but the FTA card exposed to the same mosquitoes was negative for flaviviruses [57].

The most efficacious mosquito trap in our field study was the Box gravid trap which, on average, collected over 11 times more mosquitoes within a 48-hour trapping period than the BG-Sentinel 2 trap or the BG-GAT. However, it should be noted that the BG-Sentinel 2 and BG-GAT were modified to be used in combination with the FTA cards. Perhaps these modifications have reduced the trapping success of those two trap types. Indeed, during a single trapping session the BG-Sentinel 2 ran only for about 24 hours instead of 48 hours because the fan was intermittently switched off allowing the mosquitoes to feed on the FTA cards. In a follow-up laboratory experiment we even noticed that mosquitoes were escaping from the catch bag as soon as the fan stopped because the shutter was closing too slowly to keep them inside. With regards to the BG-GATs a caveat is that they were set in pairs, separated by only 10 m. We found no noteworthy difference in mosquito count between the two BG-GAT variants which may suggest either that mosquitoes did not easily escape from both BG-GATs, regardless of the presence or absence of oil, or that they escaped at an equal rate from both traps. However, their proximity might have resulted in competition between the two BG-GATs. Nevertheless, even considering the potential bias in the number of mosquitoes caught, we still regard the Box gravid trap as the most effective trap tested as the catch rate was still a magnitude higher than in the other two traps.

In addition to being the most efficacious trap for the target species, *Ae. albopictus* and *Cx. pipiens*/*Cx. torrentium*, the Box gravid trap also caught a broader spectrum of species, including *Ae. japonicus* and *Ae. koreicus,* two additional invasive *Aedes* species, albeit this may again be linked to the larger number of mosquitoes caught. Moreover, the design of the Box gravid trap is well suited to accommodate a honey-baited FTA card because the card can conveniently be attached to the side walls of the collection chamber. Therefore, the mosquitoes can easily access the card that is protected from rain and other environmental influences. An additional advantage of the Box gravid trap is that the mosquitoes stay physically intact because they are not sucked trough the fan.

In our field trial on average 76% of the mosquitoes fed on the honey-baited FTA cards after 48 hours in the field in any of the three tested trap types. Similar feeding rates on FTA cards have been found in CO_2_-baited updraft box traps in Australia with 77%, 81% and 89% of mosquitoes being fed after 24, 72 and 168 hours, respectively [20]. In a follow-up semi-field experiment the same research group found that again 80% of females sugar-fed at least once within 72 hours in the SMACK [26]. In contrast, much lower feeding rates were observed in a field trial in Florida, USA [27]. In CO_2_-baited light traps *Ae. albopictus* feeding rates did not exceed 10% and in Box gravid traps the rate was 15%. Equally, the feeding rates for *Culex* specimens did not exceed 19% and 36%, respectively. In conclusion, when comparing our results with previous studies, the feeding rates observed here were rather high, supporting the efficacy of the approach using FTA cards in combination with traps.

The high feeding rates together with the large number of trapped mosquitoes suggest the Box gravid trap to be the optimum choice in our context. The fact that we detected USUV both in a pool of six *Cx. pipiens*/*Cx. torrentium* and on the FTA card placed in the same Box gravid trap confirms the suitability of the saliva sampling approach in combination with the Box gravid trap in an area of low prevalence. Even more so, as only two out of the six mosquitoes fed on the card as evidenced by visible blue honey in their abdomens.

The major benefit of using FTA cards for monitoring mosquito-borne viruses over extracting RNA from individuals or pools of mosquitoes is that it does not require a cold chain and is less laborious. A disadvantage of the FTA approach is that, without screening the mosquitoes in the trap, we do not know what mosquito species the virus is associated with. Still, we see the honey-baited surveillance as a cost-effective and convenient early warning tool that can be applied at large scale. Upon detection of a pathogenic virus on an FTA card the surveillance may then be complemented with targeted trapping and analysis of mosquitoes. Especially in areas with low virus circulation in mosquitoes, the approach would greatly improve if we had a marker that indicates the presence of mosquito saliva on the FTA card to rapidly exclude saliva negative FTA cards from the costly molecular screening for mosquito-borne viruses.

## Conclusions

Based on our results we conclude that honey-baited FTA cards, in combination with Box gravid traps, are an effective method for arbovirus surveillance in areas of low prevalence, particularly where resources are limited for preservation and screening of individual mosquitoes. As the approach on its own does not identify the associated mosquito vector with the virus on the FTA card we recommend to complement the approach with additional screening of mosquitoes for arboviruses in those sites where FTA cards are found to be positive.

## Supplementary information

**Additional file 1: Table S1.** Sampling schedule for the arbovirus surveillance study in Ticino 2016. **Table S2.** Mosquito trap rotation scheme.

**Additional file 2: Table S3.** Primers and probes used to detect viruses in mosquito and FTA card samples.

**Additional file 3: Text S1.** Statistical analysis in R. GLMM R code and output for mosquito trap evaluation.

**Additional file 4: Table S4.** Species composition of all collected mosquitoes including males and *Anopheles* species. **Figure S1.** Proportion of mosquito species composition in the compared trap types.

**Additional file 5: Table S5.** Raw data set.

**Additional file 6: Figure S2.** Comparison of the three trap types and number of mosquitoes that fed on the FTA card within a 48-hour trapping period. The histograms show the frequency of recorded blue mosquitoes per 48-hour trapping session (*n *= 80 for each trap type). The diamonds and horizontal bars below represent the average number of blue mosquitoes per 48-hour trapping session with 95% confidence intervals as estimated with the GLMM. **Table S6.** Mosquito feeding success on honey-baited FTA cards.

**Additional file 7: Table S7.** Summary information of virus positive samples. **Text S2.** Sequences of the four flavivirus positive samples. **Figure S3.** Amplification curves of the Usutu virus positive *Culex* (Cx-228) and FTA card (FTA-10) samples as well as the non-template control (NTC) using the USUV-specific RT-qPCR protocol.

## Data Availability

All data are presented in the text, table and figures of the article and its additional files.

## Abbreviations

BG-GAT: Biogents Gravid *Aedes* trap
BG-Sentinel 2: Biogents Sentinel 2
cDNA: complementary DNA
CHIKV: chikungunya virus
CI: confidence interval
CO_2_: carbon dioxide
DENV: dengue virus
FTA card: Whatman^™^ non-indicating Flinders Technology Associates Classic Card
GLMM: generalized linear mixed model
MOF: mosquito-only flavivirus
RNA: ribonucleic acid
RT-PCR: reverse transcription polymerase chain reaction
RT-qPCR: reverse transcription real-time polymerase chain reaction
USUV: Usutu virus
WNV: West Nile virus
ZIKV: Zika virus
