# Supplementary material for: Evaluation of honey-baited FTA cards in combination with different mosquito traps in an area of low arbovirus prevalence
Source: Parasit Vectors. 2019 Nov 21;12:554. doi: 10.1186/s13071-019-3798-8 (PMC6873520; doi:10.1186/s13071-019-3798-8)
Supplement: Supplementary file 1 — Additional file 1: Table S1. Sampling schedule for the arbovirus surveillance study in Ticino 2016. Table S2. Mosquito trap rotation scheme. [file 13071_2019_3798_MOESM1_ESM.docx]

**Additional file 1**: Sampling schedule and mosquito trap rotation scheme.

**Additional file 1: Table S1.** Sampling schedule for the arbovirus surveillance study in Ticino 2016.

| July | | | | August | | | | September | | | | October | | |
| --- | --- | --- | --- | --- | --- | --- | --- | --- | --- | --- | --- | --- | --- | --- |
| 1 | F |  |  | 1 | M |  |  | 1 | T | 2.1 Recover traps Locarnese |  | 1 | S |  |
| 2 | S |  |  | 2 | T | 1.1 Set traps Mendrisiotto |  | 2 | F |  |  | 2 | S |  |
| 3 | S |  |  | 3 | W | 1.1 Set traps Luganese |  | 3 | S |  |  | 3 | M |  |
| 4 | M |  |  | 4 | T | 1.1 Recover traps Mendrisiotto |  | 4 | S |  |  | 4 | T | 2.3 Set traps Mendrisiotto |
| 5 | T |  |  | 5 | F | 1.1 Recover traps Luganese |  | 5 | M |  |  | 5 | W | 2.3 Set traps Luganese |
| 6 | W |  |  | 6 | S |  |  | 6 | T | 1.4 Set traps Luganese |  | 6 | T | 2.3 Recover traps Mendrisiotto |
| 7 | T | Preparation of hay infusion |  | 7 | S |  |  | 7 | W | 2.2 Set traps Locarnese |  | 7 | F | 2.3 Recover traps Luganese |
| 8 | F |  |  | 8 | M | ***Reorganization of study:**  1) Optimisation BG-Sentinel 2  - 12 V, 22 Ah instead of 6 V, 10 Ah batteries  - Bottled CO_2_ instead of dry ice  2) Find replacement for field assistant |  | 8 | T | 1.4 Recover traps Luganese |  | 8 | S |  |
| 9 | S |  |  | 9 | T |  |  | 9 | F | 2.2 Recover traps Locarnese |  | 9 | S |  |
| 10 | S |  |  | 10 | W |  |  | 10 | S |  |  | 10 | M |  |
| 11 | M |  |  | 11 | T |  |  | 11 | S |  |  | 11 | T |  |
| 12 | T |  |  | 12 | F |  |  | 12 | M | 2.3 Set traps Locarnese |  | 12 | W |  |
| 13 | W | 1.1 Set traps Locarnese |  | 13 | S |  |  | 13 | T |  |  | 13 | T |  |
| 14 | T |  |  | 14 | S |  |  | 14 | W | 2.3 Recover traps Locarnese |  | 14 | F |  |
| 15 | F | 1.1 Recover traps Locarnese |  | 15 | M |  |  | 15 | T |  |  | 15 | S |  |
| 16 | S |  |  | 16 | T | 1.2 Set traps Mendrisiotto |  | 16 | F |  |  | 16 | S |  |
| 17 | S |  |  | 17 | W | 1.2 Set traps Luganese |  | 17 | S |  |  | 17 | M |  |
| 18 | M |  |  | 18 | T | 1.2 Recover traps Mendrisiotto |  | 18 | S |  |  | 18 | T |  |
| 19 | T |  |  | 19 | F | 1.2 Recover traps Luganese |  | 19 | M | 2.1 Set traps Mendrisiotto |  | 19 | W |  |
| 20 | W |  |  | 20 | S |  |  | 20 | T | 2.1 Set traps Luganese |  | 20 | T |  |
| 21 | T | 1.2 Set traps Locarnese |  | 21 | S |  |  | 21 | W | 2.1 Recover traps Mendrisiotto |  | 21 | F |  |
| 22 | F |  |  | 22 | M | 1.3 Set traps Mendrisiotto |  | 22 | T | 2.1 Recover traps Luganese |  | 22 | S |  |
| 23 | S | 1.2 Recover traps Locarnese |  | 23 | T | 1.3 Set traps Luganese |  | 23 | F |  |  | 23 | S |  |
| 24 | S |  |  | 24 | W | 1.3 Recover traps Mendrisiotto |  | 24 | S |  |  | 24 | M |  |
| 25 | M |  |  | 25 | T | 1.3 Recover traps Luganese |  | 25 | S |  |  | 25 | T |  |
| 26 | T |  |  | 26 | F |  |  | 26 | M |  |  | 26 | W |  |
| 27 | W | 1.3 Set traps Locarnese |  | 27 | S |  |  | 27 | T | 2.2 Set traps Mendrisiotto |  | 27 | T |  |
| 28 | T |  |  | 28 | S |  |  | 28 | W | 2.2 Set traps Luganese |  | 28 | F |  |
| 29 | F | 1.3. Recover traps Locarnese |  | 29 | M | 1.4 Set traps Mendrisiotto |  | 29 | T | 2.3 Recover traps Mendrisiotto |  | 29 | S |  |
| 30 | S |  |  | 30 | T | 2.1 Set traps Locarnese |  | 30 | F | 2.2 Recover traps Luganese |  | 30 | S |  |
| 31 | S |  |  | 31 | W | 1.4 Recover traps Mendrisiotto |  |  |  |  |  | 31 | M |  |

* During the first weeks of the study we observed low catch rates with the BG-Sentinel 2 trap. We intended to improve the catch rate by changing the battery type and CO_2_ source for the BG-Sentinel 2 trap. Thereafter, we decided to repeat the complete first trapping round in the districts of Mendrisiotto (Me 1.4) and Luganese (Lu 1.4). The BG-Sentinel 2 that were set in the Locarnese district were equipped with dry ice and a 6 V, 10 Ah battery for the first full rotation (Lo1.1 –Lo1.3), while they were equipped with a 12 V, 22 Ah battery for the second full rotation (Lo2.1 – Lo2.3).

Additional file 1: Table S2. Mosquito trap rotation scheme.

|  | Trap Position | Lo 1.1 | Lo 1.2 | Lo 1.3 |  | Lo 2.1 | Lo 2.2 | Lo 2.3 |
| --- | --- | --- | --- | --- | --- | --- | --- | --- |
| Locarnese | Gordola 1 | BGS^a^ | BOX | GAT |  | GAT | BGS^b^ | BOX |
|  | Gordola 2 | BOX | GAT | BGS^a^ |  | BOX | GAT | BGS^b^ |
|  | Gordola 3 | GAT | BGS^a^ | BOX |  | BGS^b^ | BOX | GAT |
|  | Tenero 1 | BGS^a^ | BOX | GAT |  | GAT | BGS^b^ | BOX |
|  | Tenero 2 | BOX | GAT | BGS^a^ |  | BOX | GAT | BGS^b^ |
|  | Tenero 3 | GAT | BGS^a^ | BOX |  | BGS^b^ | BOX | GAT |
|  | Minusio 1 | BGS^a^ | BOX | GAT |  | GAT | BGS^b^ | BOX |
|  | Minusio 2 | BOX | GAT | BGS^a^ |  | BOX | GAT | BGS^b^ |
|  | Minusio 3 | GAT | BGS^a^ | BOX |  | BGS^b^ | BOX | GAT |
|  | Locarno 1 | BGS^a^ | BOX | GAT |  | GAT | BGS^b^ | BOX |
|  | Locarno 2 | BOX | GAT | BGS^a^ |  | BOX | GAT | BGS^b^ |
|  | Locarno 3 | GAT | BGS^a^ | BOX |  | BGS^b^ | BOX | GAT |
|  |  |  |  |  |  |  |  |  |
|  | Trap Position | Me 1.1 | Me 1.2 | Me 1.3 | Me 1.4 | Me 2.1 | Me 2.2 | Me 2.3 |
| Mendrisiotto | Stabio 1 | BGS^a^ | BOX | GAT | GAT | GAT | BGS^b^ | BOX |
|  | Stabio 2 | BOX | GAT | BGS^b^ | BOX | BOX | GAT | BGS^b^ |
|  | Stabio 3 | GAT | BGS^b^ | BOX | BGS^b^ | BGS^b^ | BOX | GAT |
|  | Mendrisio 1 | BGS^a^ | BOX | GAT | GAT | GAT | BGS^b^ | BOX |
|  | Mendrisio 2 | BOX | GAT | BGS^b^ | BOX | BOX | GAT | BGS^b^ |
|  | Mendrisio 3 | GAT | BGS^b^ | BOX | BGS^b^ | BGS^b^ | BOX | GAT |
|  | Vacallo 1 | BGS^a^ | BOX | GAT | GAT | GAT | BGS^b^ | BOX |
|  | Vacallo 2 | BOX | GAT | BGS^b^ | BOX | BOX | GAT | BGS^b^ |
|  | Vacallo 3 | GAT | BGS^b^ | BOX | BGS^b^ | BGS^b^ | BOX | GAT |
|  | Chiasso 1 | BGS^a^ | BOX | GAT | GAT | GAT | BGS^b^ | BOX |
|  | Chiasso 2 | BOX | GAT | BGS^b^ | BOX | BOX | GAT | BGS^b^ |
|  | Chiasso 3 | GAT | BGS^b^ | BOX | BGS^b^ | BGS^b^ | BOX | GAT |
|  |  |  |  |  |  |  |  |  |
|  | Trap Position | Lu 1.1 | Lu 1.2 | Lu 1.3 | Lu 1.4 | Lu 2.1 | Lu 2.2 | Lu 2.3 |
| Luganese | Pregassona 1 | BGS^a^ | BOX | GAT | BGS^b^ | GAT | BGS^b^ | BOX |
|  | Pregassona 2 | BOX | GAT | BGS^b^ | BOX | BOX | GAT | BGS^b^ |
|  | Pregassona 3 | GAT | BGS^b^ | BOX | GAT | BGS^b^ | BOX | GAT |
|  | Lugano 1 | BGS^a^ | BOX | GAT | BGS^b^ | GAT | BGS^b^ | BOX |
|  | Lugano 2 | BOX | GAT | BGS^b^ | BOX | BOX | GAT | BGS^b^ |
|  | Lugano 3 | GAT | BGS^b^ | BOX | GAT | BGS^b^ | BOX | GAT |
|  | Massagno 1 | BGS^a^ | BOX | GAT | BGS^b^ | GAT | BGS^b^ | BOX |
|  | Massagno 2 | BOX | GAT | BGS^b^ | BOX | BOX | GAT | BGS^b^ |
|  | Massagno 3 | GAT | BGS^b^ | BOX | GAT | BGS^b^ | BOX | GAT |
|  | Paradiso 1 | BGS^a^ | BOX | GAT | BGS^b^ | GAT | BGS^b^ | BOX |
|  | Paradiso 2 | BOX | GAT | BGS^b^ | BOX | BOX | GAT | BGS^b^ |
|  | Paradiso 3 | GAT | BGS^b^ | BOX | GAT | BGS^b^ | BOX | GAT |

| BGS^a^ | BG-Sentinel 2 baited with dry ice and powered with a 6 V, 10 Ah lead-acid battery* |
| --- | --- |
| BGS^b^ | BG-Sentinel 2 baited with bottled CO_2_ and powered with a 12 V, 22 Ah lead-acid battery* |
| GAT | Two variants of BG-GAT set in pairs at a distance of at least 10 m |
| BOX | Box gravid trap |
| Me 1.4 | Repetition of Me1.1, however trap position 1 and 3 were interchanged |
| Lu 1.4 | Repetition of Lu1.1 |

*There was no significant difference in catch rate between the BGS^a^ and BGS^b^ (*χ^2^* =7.35, *df*=7, *n*=80, *P*=0.39).
All trappings were included in the analysis.
