# Supplementary material for: Evaluation of honey-baited FTA cards in combination with different mosquito traps in an area of low arbovirus prevalence
Source: Parasit Vectors. 2019 Nov 21;12:554. doi: 10.1186/s13071-019-3798-8 (PMC6873520; doi:10.1186/s13071-019-3798-8)
Supplement: Supplementary file 2 — Additional file 2: Table S3. Primers and probes used to detect viruses in mosquito and FTA card samples. [file 13071_2019_3798_MOESM2_ESM.docx]

Additional file 2: Table S3. Primers and probes used to detect viruses in mosquito and FTA card samples.

| PCR assay | Primer/Probe Name^a^ | Oligonucleotide sequence (5’- 3’) | Amplicon length | Reference |
| --- | --- | --- | --- | --- |
| Mengovirus RT-qPCR | Mengo F2 | TGCCAACCCAAAACCACAT | NA | Costafreda et al. 2006 [32] |
|  | Mengo R2 | ACGCACACCGCCTTATTC |  |  |
|  | Mengo P1 | FAM-CTCACATTACTGGCCGAAGCCGCT-BHQ1 |  |  |
|  |  |  |  |  |
| PanAlpha nested  RT-PCR | Alpha 1+ F | GAYGCITAYYTIGAYATGGTIGAIGG | 481 bp | Sánchez-Seco et al. 2001 [33] |
|  | Alpha 1- R | KYTCYTCIGTRTGYTTIGTICCIGG |  |  |
|  | Alpha 2+ F | GIAAYTGYAAYGTIACICARATG | 195 bp |  |
|  | Alpha 2- R | GCRAAIARIGCIGCIGCYTYIGGICC |  |  |
|  |  |  |  |  |
| PanFlavi semi-nested  RT-PCR | MAMD F | AACATGATGGGRAARAGRGARAA | 263 bp 215 bp | Scaramozzino et al. 2001 [34] |
|  | cFD2 R | GTGTCCCAGCCGGCGGTGTCATCAGC |  |  |
|  | FS778 F | AARGGHAGYMCDGCHATHTGGT |  |  |
|  |  |  |  |  |
| Usutu virus  RT-qPCR | UsuE F2 | CAAGCTCACATCTGGTCATCTCA | NA | in-house**^b^** |
|  | UsuE R2 | GTCAGCCGGATTTTTTGCAA |  |  |
|  | UsuE P2 | FAM-AAGTTGACACTAAAAGGCACCACCTACGGC-BHQ1 |  |  |

^a^ F: forward primer; R: reverse primer; P: TaqMan™ probe (FAM fluorophore, BHQ1 quencher)
^b^ Usutu virus specific in-house protocol of the Spiez Laboratory, Switzerland that was used the same RT-qPCR reagent volumes, primer concentrations and
cycling conditions as for the mengovirus-specific RT-qPCR protocol described in the methods section of the main article.
