# Supplementary material for: Evaluation of honey-baited FTA cards in combination with different mosquito traps in an area of low arbovirus prevalence
Source: Parasit Vectors. 2019 Nov 21;12:554. doi: 10.1186/s13071-019-3798-8 (PMC6873520; doi:10.1186/s13071-019-3798-8)
Supplement: Supplementary file 3 — Additional file 3: Text S1. Statistical analysis in R. GLMM R code and output for mosquito trap evaluation. [file 13071_2019_3798_MOESM3_ESM.docx]

**Additional file 3: Text S1.** Statistical analysis in R. GLMM R code and output for mosquito trap evaluation.

| variable | description |
| --- | --- |
| **count** | Total number of *Aedes* and *Culex* females counted in each trap (after 48 h trapping) |
| **N_blue** | Number of blue *Ae.* and *Cx.* females counted in each trap (fed on FTA card) |
| **N_not_blue** | Number of *Ae.* and *Cx.* females in each trap that were not blue (not fed on FTA card) |
| **trap** | Variable for either of the four trap types (BGS, BOX, GAT_FTA and GAT_OIL) |
| BGS | BG-Sentinel 2 trap = (Intercept) |
| BOX | Box gravid trap |
| GAT_FTA | BG-GAT with honey-baited FTA card taped to the untreated translucent chamber |
| GAT_OIL | BG-GAT translucent chamber with a film of canola oil (without FTA card) |
| **position*** | trap position (*n* = 36) ~ geographical point |
| **col_code*** | collection codes (*n* = 20) ~ date of collection (6 trapping rounds in Locarnese, 7 trapping rounds in Mendrisiotto and Luganese) |

(*see Additional file 1: Table S2)

1. **GLMM to evaluate the most efficacious mosquito trap type in our study setting**


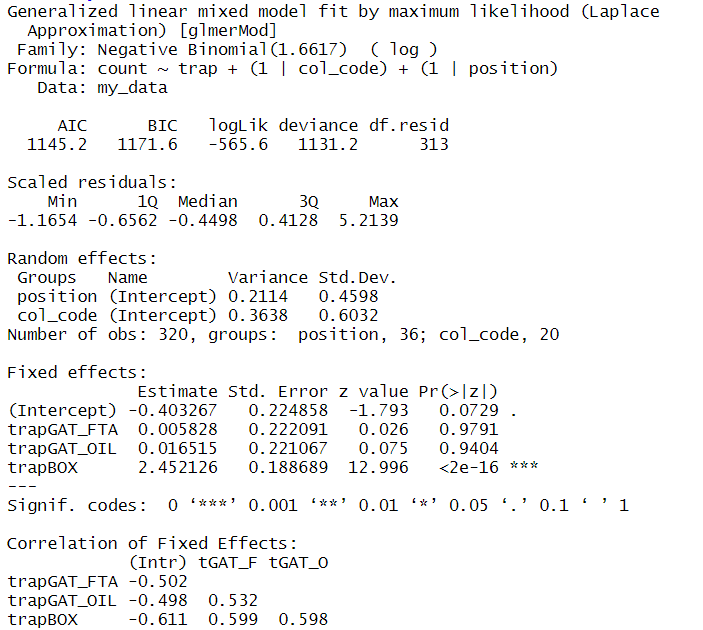


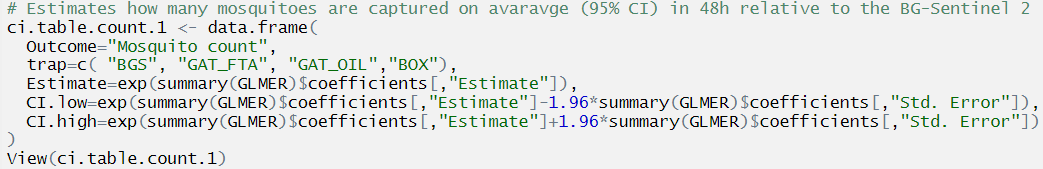


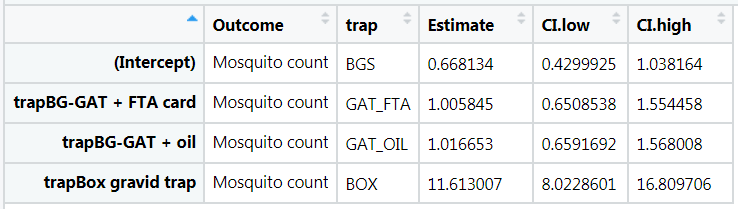


Both, adding a zero-inflation parameter (to account for the excessive number of zero counts) or a random effect for a new variable with a different factor for each observation (to control for an unknown source of variation), did not improve the model fit. Nor did including the variables “district” and/or “municipality” as a random effect term.

The average mosquito count per trap type and the 95% confidence intervals (CIs) were calculated as the exponential of the estimate coefficient of the same model without intercept.
R code: glmer.nb(count ~ 0 + trap + (1|col_code) + (1|position).


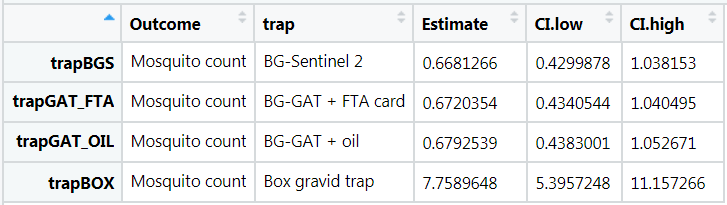


1. **Evaluation of average proportion of sugar-feeding on FTA cards**


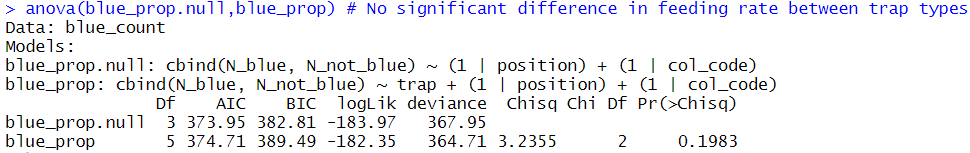


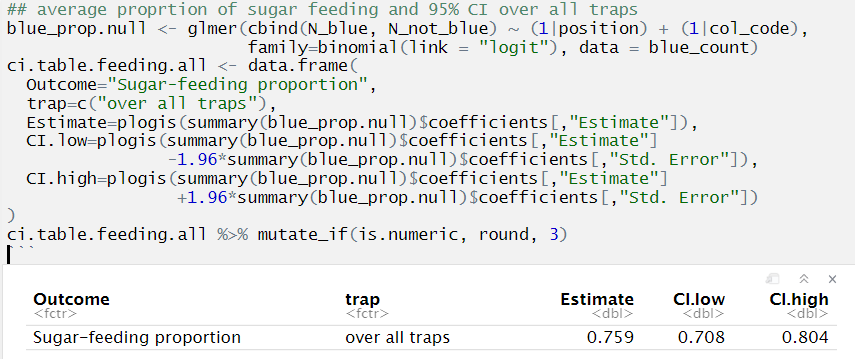


1. **GLMM for average number of sugar fed mosquitoes per 48-hour trapping period**


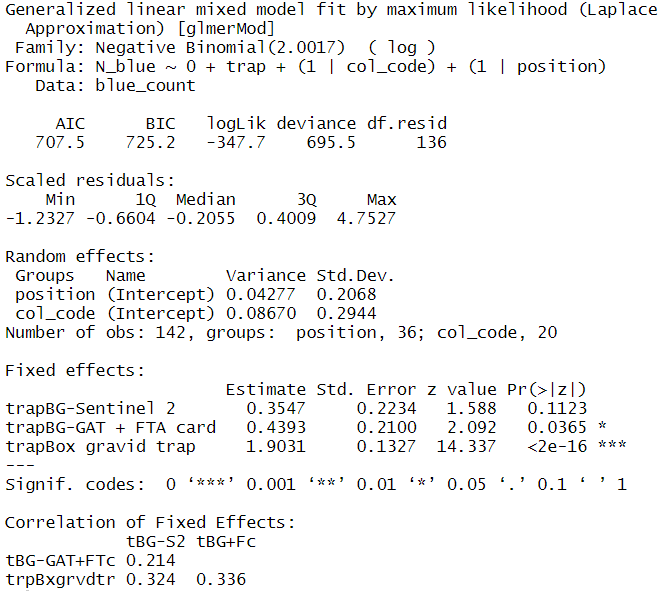


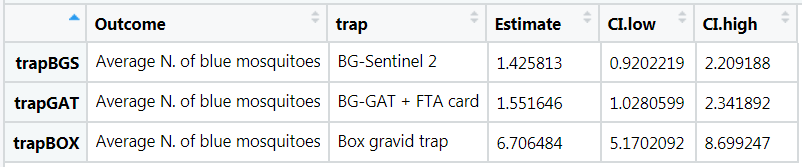


See Additional file 6: Figure S2 for visualisation plot.
