# Supplementary material for: Evaluation of honey-baited FTA cards in combination with different mosquito traps in an area of low arbovirus prevalence
Source: Parasit Vectors. 2019 Nov 21;12:554. doi: 10.1186/s13071-019-3798-8 (PMC6873520; doi:10.1186/s13071-019-3798-8)
Supplement: Supplementary file 4 — Additional file 4: Table S4. Species composition of all collected mosquitoes including males and Anopheles species. Figure S1. Proportion of mosquito species composition in the compared trap types. [file 13071_2019_3798_MOESM4_ESM.docx]

**Additional file 4:** Species composition of all collected mosquitoes.

Additional file 4: Table S4 Species composition of all collected mosquitoes including males and *Anopheles* species.

|  |  | | | Mosquito trap | | | | | | | | | | | |
| --- | --- | --- | --- | --- | --- | --- | --- | --- | --- | --- | --- | --- | --- | --- | --- |
|  | Overall study collection | | | BG-Sentinel 2 | | | BG-GAT with FTA card | | | BG-GAT with oil | | | Box Gravid Trap | | |
| Species | Total N. (%) | f | m | Total N. (%) | f | m | Total N. (%) | f | m | Total N. (%) | f | m | Total N. (%) | f | m |
| *Aedes*  *albpopictus* | 666 (47.5) | 467 | 199 | 57 (57.6) | 36 | 21 | 76 (85.4) | 62 | 14 | 88 (88.9) | 68 | 20 | 445 (39.9) | 301 | 144 |
| *Aedes*  *geniculatus* | 1 (0.1) | 1 | 0 | 0 | 0 | 0 | 0 | 0 | 0 | 0 | 0 | 0 | 1  (0.1) | 1 | 0 |
| *Aedes*  *japonicus* | 26 (1.9) | 25 | 1 | 0 | 0 | 0 | 2  (2.2) | 2 | 0 | 3  (3.0) | 3 | 0 | 21 (1.9) | 20 | 1 |
| *Aedes*  *koreicus* | 17 (1.2) | 14 | 3 | 0 | 0 | 0 | 0 | 0 | 0 | 0 | 0 | 0 | 17 (1.5) | 14 | 3 |
| *Anopheles*  *maculipennis s.l.* | 2 (0.1) | 2 | 0 | 1 (1.0) | 1 | 0 | 0 | 0 | 0 | 0 | 0 | 0 | 1  (0.1) | 1 | 0 |
| *Anopheles*  *plumbeus* | 8 (0.6) | 5 | 3 | 0 | 0 | 0 | 0 | 0 | 0 | 1 (1.0) | 1 | 0 | 7 (0.6) | 4 | 3 |
| *Culex*  *hortensis* | 35 (2.5) | 23 | 12 | 0 | 0 | 0 | 0 | 0 | 0 | 0 | 0 | 0 | 35 (3.1) | 23 | 12 |
| *Culex*  *pipiens/ torrentium* | 645 (46) | 477 | 168 | 41  (41.4) | 35 | 6 | 11 (12.4) | 10 | 1 | 6 (6.1) | 6 | 0 | 587 (52.7) | 426 | 161 |
| undetermined species | 1 (0.1) | 1 | 0 | 0 | 0 | 0 | 0 | 0 | 0 | 1 (1.0) | 1 | 0 | 0 | 0 | 0 |
| Total | 1401 | 1015 | 386 | 99 | 72 | 27 | 89 | 74 | 15 | 99 | 79 | 20 | 1114 | 790 | 324 |

Absolute numbers of females (f) and males (m) and their sum (and percentage of a particular species per category in brackets) are recorded for the overall study
and for each trap type.


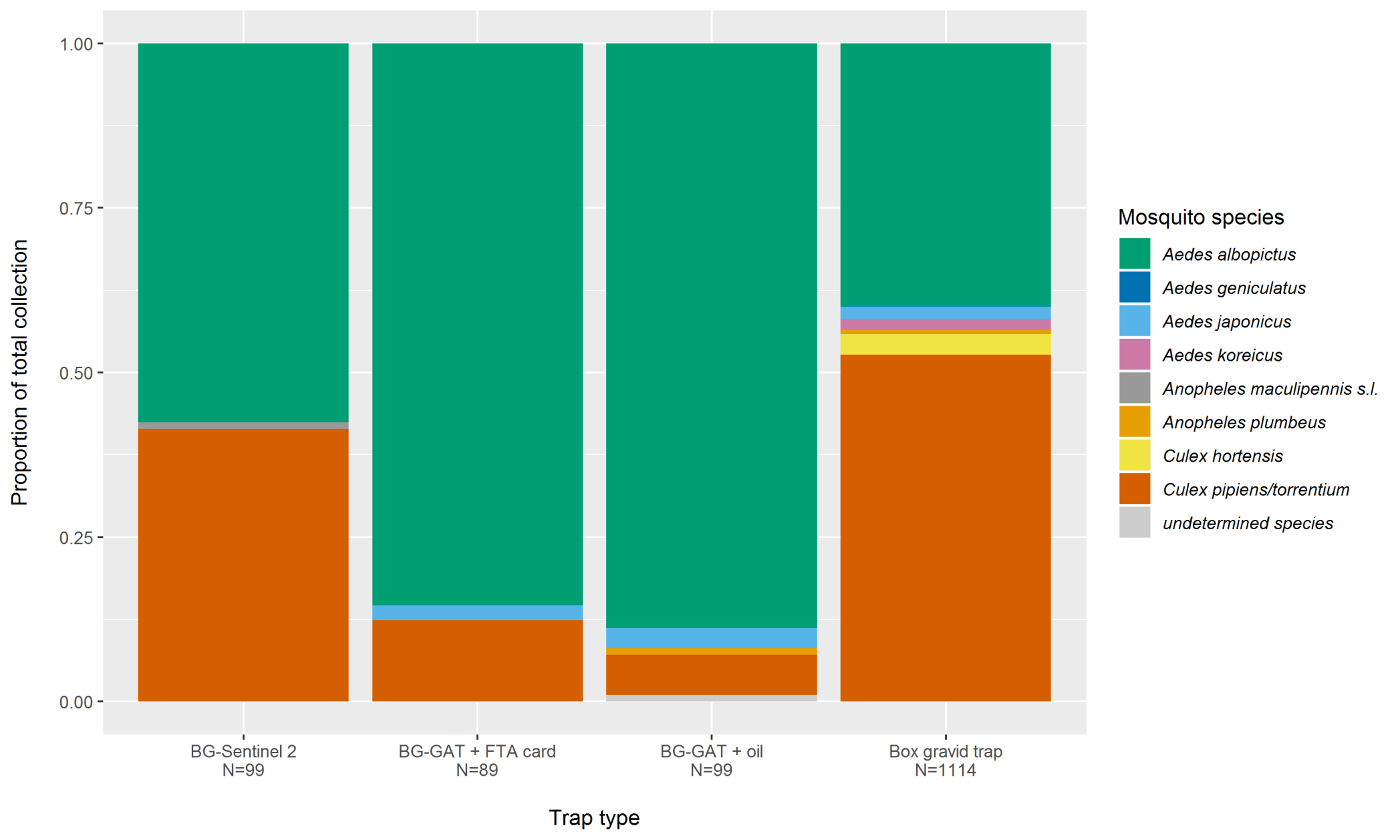


Additional file 4: Figure S1 Proportion of mosquito species composition in the compared trap types.
