# Supplementary material for: Evaluation of honey-baited FTA cards in combination with different mosquito traps in an area of low arbovirus prevalence
Source: Parasit Vectors. 2019 Nov 21;12:554. doi: 10.1186/s13071-019-3798-8 (PMC6873520; doi:10.1186/s13071-019-3798-8)
Supplement: Supplementary file 6 — Additional file 6: Figure S2. Comparison of the three trap types and number of mosquitoes that fed on the FTA card within a 48-hour trapping period. The histograms show the frequency of recorded blue mosquitoes per 48-hour trapping session (n = 80 for each trap type). The diamonds and horizontal bars below represent the average number of blue mosquitoes per 48-hour trapping session with 95% confidence intervals as estimated with the GLMM. Table S6. Mosquito feeding success on honey-baited FTA cards. [file 13071_2019_3798_MOESM6_ESM.docx]

**Additional file 6:** Mosquito feeding success on honey-baited FTA cards.


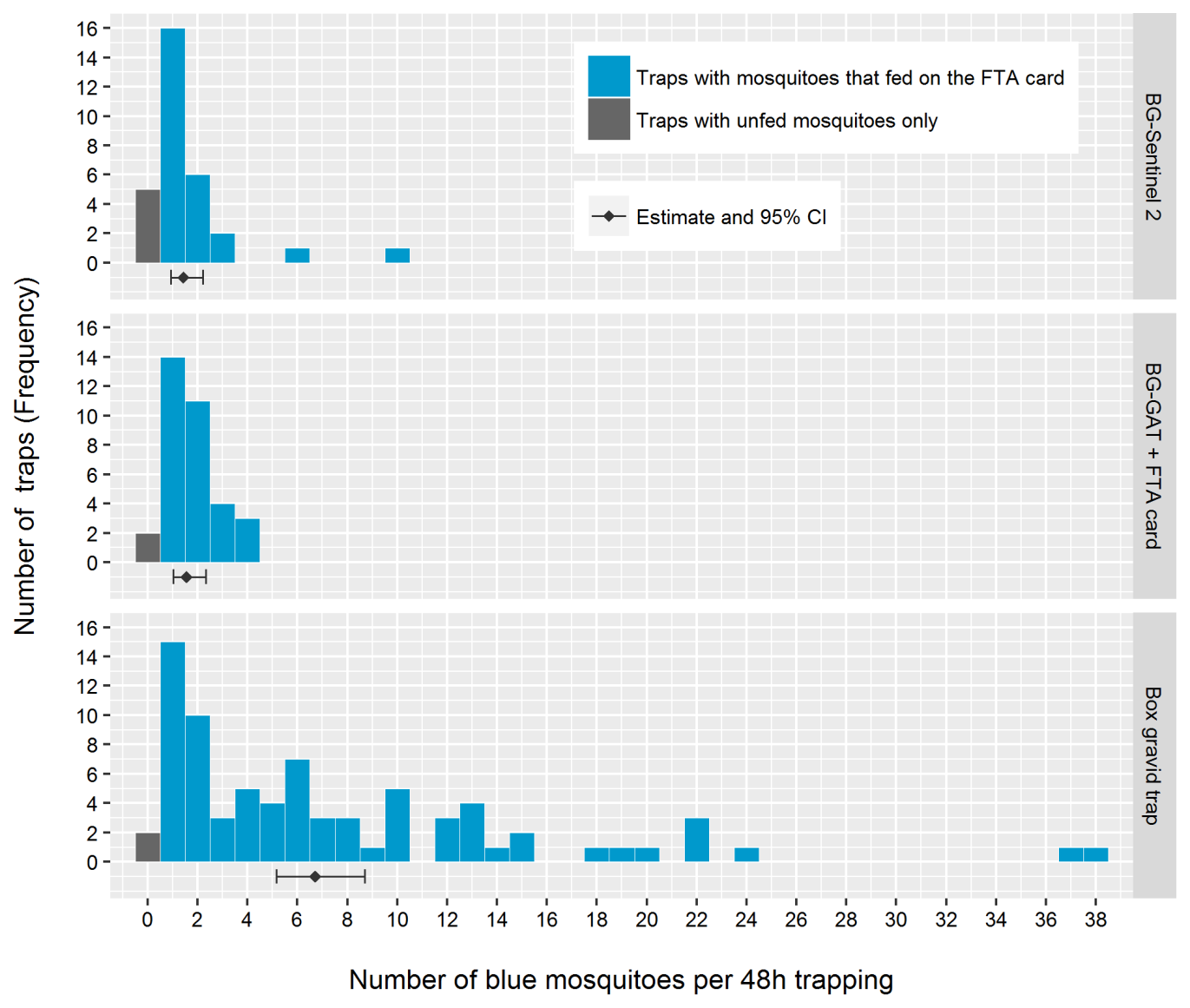


Additional file 6: Figure S2. Comparison of the three trap types and number of mosquitoes that fed on the FTA card within a 48-hour trapping period. The histograms show the frequency of recorded blue mosquitoes per 48-hour trapping session (*n* = 80 for each trap type). The diamonds and horizontal bars below represent the average number of blue mosquitoes per 48-hour trapping session with 95% confidence intervals as estimated with the GLMM. See outcome below and Additional file 3: Text S1 for R code.


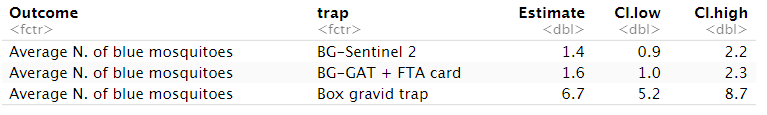


Additional file 6: Table S6. Mosquito feeding success on honey-baited FTA cards.

|  | Number of blue mosquitoes / total (% fed on FTA card) | | |
| --- | --- | --- | --- |
|  | Total | f | m |
| BG-Sentinel 2 | 65 / 99  (65.7%) | 51 / 72  (70.8%) | 14 / 27  (51.9%) |
| BG-GAT with FTA card | 74 / 89  (83.1%) | 60 / 74  (81.1%) | 14 / 15  (93.3%) |
| Box Gravid Trap | 821 / 1114 (73.7%) | 585 / 790  (74.1%) | 236 / 324  (72.8%) |
| Overall study collation | 960 / 1302 (73.7%) | 696 / 936  (74.4%) | 264 / 366  (72.1%) |

Absolute numbers and percentages of female (f) and male (m) mosquitoes and their sum are recorded for each trap type and in the overall study. Chi-square test of independence revealed that there was no significant difference in sugar feeding rates between female and male mosquitoes (*χ^2^*=0.56, *df*=1, *n*=1302, *P*=0.45).
