# Supplementary material for: Evaluation of honey-baited FTA cards in combination with different mosquito traps in an area of low arbovirus prevalence
Source: Parasit Vectors. 2019 Nov 21;12:554. doi: 10.1186/s13071-019-3798-8 (PMC6873520; doi:10.1186/s13071-019-3798-8)
Supplement: Supplementary file 7 — Additional file 7: Table S7. Summary information of virus positive samples. Text S2. Sequences of the four flavivirus positive samples. Figure S3. Amplification curves of the Usutu virus positive Culex (Cx-228) and FTA card (FTA-10) samples as well as the non-template control (NTC) using the USUV-specific RT-qPCR protocol. [file 13071_2019_3798_MOESM7_ESM.docx]

**Additional file 7:** Summary information, sequences and RT-qPCR amplification curves of virus positive samples.

Additional file 7: Table S7. Summary information of virus positive samples.

| Sample ID | Sample type (mosquito species / FTA card) | No. total pool | No. blue | Virus detected | District | Collection code | Trap position | Trap type | Set date | Reco-very date |
| --- | --- | --- | --- | --- | --- | --- | --- | --- | --- | --- |
| **Ae-159** | *Aedes albopictus* (f) | 3 | NA | MOF | Locarnese | Lo2.2 | TEN2 | GAT+OIL | 2016-09-07 | 2016-09-09 |
| **Ae-188** | *Aedes albopictus* (f) | 1 | 1 | MOF | Mendrisiotto | Me2.1 | STA2 | BOX | 2016-09-19 | 2016-09-21 |
| **Cx-228** | *Culex pipiens/ torrentium* (f) | 6 | 2 | USUV | Mendrisiotto | Me2.2 | VAC3 | BOX | 2016-09-27 | 2016-09-29 |
| **FTA-10** | FTA card | NA | NA | USUV | Mendrisiotto | Me2.2 | VAC3 | BOX | 2016-09-27 | 2016-09-29 |

**Additional file 7:** **Text S2.** Sequences of the four flavivirus positive samples. After sequencing the PanFlavi RT-PCR products in both directions, we aligned the two obtained sequences for each sample and trimmed them of low quality. We submitted the complete sequences to GenBank (https://www.ncbi.nlm.nih.gov/).

>Seq1 [organism=Mosquito flavivirus] [isolate=MOF_Aa-159_TI2016] non-structural protein 5 (NS5) gene, partial cds

GenBank accession number Seq1: MN566100

TGGTATATGTGGCTCGGGAGCAGATTCTTGGAGTACGAAGCGCTGGGGTTCCTGAACGCAGACCACTGGGTCAGCCGTGAGAATTTCCCCGGAGGAGTTGGCGGTGTTGGCGTCAACTATTTTGGCAATTATCTCAAGGAGATTTCCTGCAAAGGAAAGTACCTCTTTGCTGATGACACCGCCGGCTGG

>Seq2 [organism=Mosquito flavivirus] [isolate=MOF_Aa-188_TI2016] non-structural protein 5 (NS5) gene, partial cds

GenBank accession number Seq2: MN566101

TGGTATATGTGGCTCGGGAGCAGATTCTTGGAGTACGAAGCGCTGGGGTTCCTGAACGCAGACCACTGGGTCAGCCGTGAGAATTTCCCCGGAGGAGTTGGCGGTGTTGGCGTCAACTATTTTGGCAATTATCTCAAGGAGATTTCCTGCAAAGGAAAGTACCTCTTTGCTGATGACACCGCCGGCTGGGACACA

>Seq3 [organism=Usutu virus] [isolate=USUV_Cx-228_TI2016] non-structural protein 5 (NS5) gene, partial cds

GenBank accession number Seq3: MN566102

TGGTTCATGTGGCTAGGCGCCAGATTCCTGGAGTTTGAAGCCCTGGGCTTTCTGAATGAGGACCATTGGTTAGGAAGAAAGAATTCTGGAGGAGGTGTTGAAGGACTTGGTGTCCAAAAACTTGGTTACATTCTGCGTGAGATGAGCCACCATTCAGGTGGAAAAATGTACGCTGATGACACCGCCGGCTGGGACACA

>Seq4 [organism=Usutu virus] [isolate=USUV_FTA-10_TI2016] non-structural protein 5 (NS5) gene, partial cds

GenBank accession number Seq4: MN566103

TGGTTCATGTGGCTAGGCGCCAGATTCCTGGAGTTTGAAGCCCTGGGCTTTCTGAATGAGGACCATTGGTTAGGAAGAAAGAATTCTGGAGGAGGTGTTGAAGGACTTGGTGTCCAAAAACTTGGTTACATTCTGCGTGAGATGAGCCACCATTCAGGTGGAAAAATGTACGCTGATGACACCGCCGGCTGGGACACA


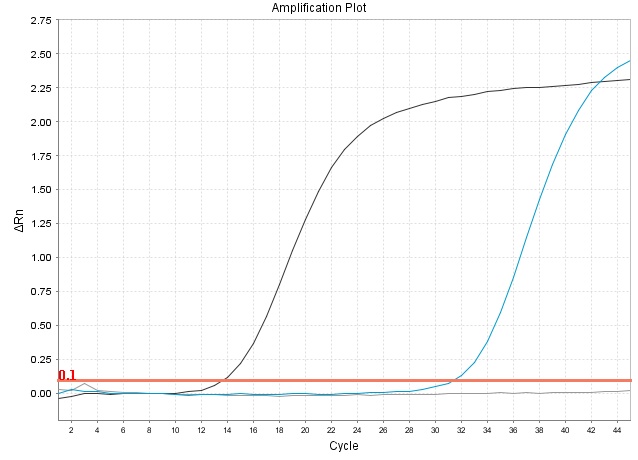


Cx-228
Cq = 13.7

FTA-10
Cq = 31.5

NTC

Additional file 7: Figure S3. Amplification curves of the Usutu virus positive *Culex* (Cx-228) and
FTA card (FTA-10) samples as well as the non-template control (NTC) using the USUV-specific
RT-qPCR protocol.
